# Cochlear Sox2^+^ Glial Cells Are Potent Progenitors for Spiral Ganglion Neuron Reprogramming Induced by Small Molecules

**DOI:** 10.3389/fcell.2021.728352

**Published:** 2021-09-21

**Authors:** Zhen Chen, Yuhang Huang, Chaorong Yu, Qing Liu, Cui Qiu, Guoqiang Wan

**Affiliations:** ^1^MOE Key Laboratory of Model Animal for Disease Study, Department of Otorhinolaryngology Head and Neck Surgery, The Affiliated Drum Tower Hospital of Medical School, Model Animal Research Center of Medical School, Nanjing University, Nanjing, China; ^2^Research Institute of Otolaryngology, Nanjing, China; ^3^Jiangsu Key Laboratory of Molecular Medicine, Medical School of Nanjing University, Nanjing, China; ^4^Institute for Brain Sciences, Nanjing University, Nanjing, China

**Keywords:** Sox2^+^ glial cells, glia-to-neuron conversion, small molecules reprogramming, SGN regeneration, lineage tracing

## Abstract

In the mammalian cochlea, spiral ganglion neurons (SGNs) relay the acoustic information to the central auditory circuits. Degeneration of SGNs is a major cause of sensorineural hearing loss and severely affects the effectiveness of cochlear implant therapy. Cochlear glial cells are able to form spheres and differentiate into neurons *in vitro*. However, the identity of these progenitor cells is elusive, and it is unclear how to differentiate these cells toward functional SGNs. In this study, we found that Sox2^+^ subpopulation of cochlear glial cells preserves high potency of neuronal differentiation. Interestingly, Sox2 expression was downregulated during neuronal differentiation and Sox2 overexpression paradoxically inhibited neuronal differentiation. Our data suggest that Sox2^+^ glial cells are potent SGN progenitor cells, a phenotype independent of Sox2 expression. Furthermore, we identified a combination of small molecules that not only promoted neuronal differentiation of Sox2^–^ glial cells, but also removed glial cell identity and promoted the maturation of the induced neurons (iNs) toward SGN fate. In summary, we identified Sox2^+^ glial subpopulation with high neuronal potency and small molecules inducing neuronal differentiation toward SGNs.

## Introduction

In the mammalian cochlea, the spiral ganglion neurons (SGNs) relay the acoustic information from inner hair cells (IHCs) to the central auditory circuits (Fettiplace, 2017). SGNs are essential for normal hearing and communication, and degeneration causes sensorineural hearing loss (Sun et al., 2016; Liu et al., 2019; Zhao et al., 2019). Degeneration of SGN nerve terminals or cell bodies can be caused by ototoxicity, noise, or aging (Lang et al., 2005; Bao and Ohlemiller, 2010; Fryatt et al., 2011; Liberman, 2017; Vlajkovic et al., 2017). Because the SGNs lack the ability to regenerate in mammals, damages to the SGNs lead to permanent hearing impairment (Guo et al., 2016, 2020, 2021; Yan et al., 2018; Liu et al., 2021). In addition, the effectiveness of hearing aids and cochlear implants relies on the health and numbers of intact SGNs (Muller and Barr-Gillespie, 2015). If SGNs could be replaced or regenerated, it might be possible to restore the hearing of patients with severely damaged SGNs (Meas et al., 2018b) and benefit individuals treated with hearing aids and cochlear implants.

At the early stage of SGN damage which precedes neuronal cell body degeneration, neurotrophic factors such as neurotrophin 3 (NT3), brain-derived neurotrophic factor (BDNF), and glial-derived neurotrophic factor (GDNF) are used to support the survival of SGNs and their neurite outgrowth to the sensory HCs (Wise et al., 2011; Suzuki et al., 2016; Akil et al., 2019). However, therapeutic strategies are limited to generate induced neurons (iNs) to replace the SGNs once they are lost and SGN regeneration remains a major challenge.

Multiple attempts have been made to replace and regenerate SGNs, including transplantations of iNs differentiated from embryonic stem cells (ESC) or iPSC-derived progenitors (Chen et al., 2012; Koehler et al., 2013; Perny et al., 2017), or neuronal differentiation of cochlear-resident multipotent stem cells/progenitor cells to SGNs (Oshima et al., 2007; Zhang et al., 2011; Diensthuber et al., 2014a,b; Li et al., 2016; McLean et al., 2016; Noda et al., 2018). For induced differentiation of ESC or iPSC-derived progenitors, three-dimensional culture systems have been used to convert mouse ESC into hair cells, supporting cells, and neuronal cells (Koehler et al., 2013; Perny et al., 2017). hESC differentiated into neuronal cells expressed specific neuronal markers with electrophysiological properties characteristic of auditory neurons (Chen et al., 2012). However, transplantation of ESC-derived iNs is hampered by immuno-rejection, tumorigenesis, SGN maturation and functional integration (Lee et al., 2013; Lukovic et al., 2014).

Alternatively, inner ear-resident cells, such as progenitor cells within the utricle (Li et al., 2003) or in the spiral ganglion region (Oshima et al., 2007; Zhang et al., 2011; Li et al., 2016; McLean et al., 2016), could also be induced to neuron-like cells, forming neurites, developing synapses and expressing neuronal markers *in vitro*. It has been reported that Plp1^+^ glial cells were cochlear-resident multipotent stem cells/progenitor cells (McLean et al., 2016). Induced neuronal reprogramming of these cochlear-resident progenitors have several advantages over cell transplantations such as enhanced cell survival, physiological relevance of cellular localization and ease of maturation due to lineage similarities. However, Plp1^+^ glial cells may include heterogenous cell subpopulation. The identity of these progenitor cells is elusive, and it is unclear how to induce these cells to functional SGNs post-injury both *in vitro* and *in vivo*.

In this study, we found that Sox2^+^ subpopulation of cochlear glial cells preserves high potency of neuronal differentiation and identified a combination of small molecules promoted the maturation of the iNs toward SGNs fate. Together, we found that cochlear Sox2^+^ glial cells subpopulation is highly potent in neuronal differentiation and identified small molecules promoting both neuronal differentiation efficiency and maturity toward the SGN fate.

## Materials and Methods

### Animals

Plp1^CreERT^ (stock number 005975), Sox2^CreERT^, (stock number 017593) lines were obtained from Jackson laboratory and crossed with Rosa26-LSL-Cas9-tdTomato (NBRI T002249) mice obtained from Gempharmatech Inc., China. Scrt2-P2A-tdTomato mice were generated as previously reported (Li C. et al., 2020). Mice were injected i.p. with Tamoxifen (Sigma, T5648) at 33 mg/kg for postnatal mice (P1–P3) and 50 mg/kg for juvenile mice (P17–P20). Tamoxifen was dissolved in corn oil.

All mice used in this work were on a mixed background containing C57BL6 and FVB/N strains. Both male and female mice were used. All animal procedures were approved by the Institutional Animal Care and Use Committee of Model Animal Research Center of Nanjing University.

### Cell Culture

Both sexes of the 6–8 postnatal 4–21 days (P4–P21) mice spiral ganglia were dissected in HBSS (Invitrogen 14065056) at pH 7.4 on ice for tissue harvesting. The stria vascularis, vestibule and the organ of Corti were removed carefully with forceps (Dumont) to dissect the modiolus. The modiolus were digested with Trypsin/EDTA (Sigma 59418C) and DNase I (20 U/ml) at 37°C in a total volume of 50 μl for ∼15 min with shaking at 300 rpm/min (Thermo) in 1.5 ml EP tube. Dissociation was terminated by adding 0.4 ml SCM media containing DMEM/F12 (HyClone 36254) supplemented with B27 (Thermo 17504044) and N2 (Thermo 17502048) supplement, 20 ng/ml EGF (Peprotech 315-09), 10 ng/ml bFGF (Peprotech 450-33), 50 ng/ml IGF (Peprotech 250-19), and 50 ng/ml heparan sulfate (MCE HY-101916). The samples were then carefully triturated with 1 ml pipette tips and next with 200 μl tips, followed by suspension with 1 ml SCM medium.

The cell suspension was then passed through a 70-μm cell strainer, and spun at 300 g for 5–10 min. A small white cell pellet should be observed at the bottom and then carefully aspirate the supernatant. SCM medium were added to resuspend and count cells, and then 200,000–240,000 cells were plated in each well of the six-well dish (Corning 3471). For propagation, the cochlear glial spheres were harvested after 5–7 days and passaged for 3–4 generations.

For P21 mice, the modiolus were calcified and the cochlear glial cells decrease the potential to form spheres. P21 modiolus were digested with Trypsin/EDTA (Sigma 59418C) and DNase I (20 U/ml) at 37°C in a total volume of 50–100 μl for ∼20 min with shaking 300 rpm/min (Thermo) in 1.5 ml EP tube. We used culture media contains DMEM/F12, 10% FBS, B27 in 2D dish (Lang et al., 2011). Firstly, culture media was added to stop trypsin reaction. Tissues were triturated with pipet tips and centrifuged at 300 × *g* for 5–10 min. The pellet was resuspended in culture media and filtered through a 70 μm cell strainer. Cells were counted, plated and grown to full confluency (5–7 days). Media was then removed and replaced with SCM media for suspension culture.

### Neuronal Differentiation

To induce neuronal differentiation, cochlear glial spheres were plated on 96-well plate (Thermo 310109008) or glass slides (Thermo Fisher 12-545-80) coated with Poly-L-ornithine (Sigma P4957) and 10 ng/ml Laminin (Corning 354232) in SCM for 12–24 h, and then replaced with SCDM containing DMEM/F12 (HyClone 36254) supplemented with B27 (Thermo 17504044) and N2 (Thermo 17502048) supplement, 50 ng/ml BDNF (Stemcell 78005), 50 ng/ml NT3 (Stemcell 78074). Half of the medium was replaced every 2–3 days. Differentiated cells were analyzed after 9 days or more for immunocytochemistry and qPCR. Additional control SCDM/FGF referred to SCDM supplemented with bFGF (100 ng/ml).

The induction media (IM) for small molecule reprogramming contains Neurobasal Medium (Thermo 21103049), supplemented with B27 and N2, GlutaMax (Thermo 35050061), penicillin-streptomycin and bFGF (100 ng/ml), with or without small molecules Forskolin (20 μM), ISX9 (20 μM), I-BET (1–2 μM), Chir99021 (10 μM) (all from Selleck), and LIF (1000 U/ml) (Novus Biologicals).

### Real-Time Quantitative PCR

RNA was isolated using Trizol (Takara 9108) and reverse transcription of total RNA was performed with the Primescript RT reagent kit (Takara RR047A) according to the manufacturer’s protocol. The Quantitative PCR reactions were performed with the Hieff UNICON^®^ qPCR SYBR Green Master Mix (YEASEN 11198ES03) on LightCycler 96 (Roche LightCycler^®^ 96 Instrument). Details of the primers were in Table 1. Data are normalized to GAPDH, and fold changes are calculated by using 2^–ΔΔCT^ method.

**TABLE 1:** Primers used for real-time qPCR.

Gene ID	Forward (5′–3′)	Reverse (5′–3′)
*GAPDH*	ACCACGAGAAATATGACAAC TCAC	CCAAAGTTGTCATGGATGACC
*Tubb3*	TAGACCCCAGCGGCAACTAT	GTTCCAGGTTCCAAGTCCACC
*Nestin*	ACAGTGAGGCAGATGAGTTAGG	GAGGCAGGAGACTTCAGGTAG
*Prox1*	TCTCAGCCAAACCCTCTC	CCGTTGACTGCGAATCTG
*Sox2*	GCGGAGTGGAAACTTTTGTCC	CGGGAAGCGTGTACTTATCCTT
*Vglut1*	GTTCTGGCTTCTGGTGTCT TATG	CTCTCCAATGCTCTCCTCTATGT
*Islet1*	CTTGCGGACCTGCTATGC	AACCACACTCGGATGACTCT
*Scrt2*	GTCCTCTGCCTGTCCATTCCT	GCTGCCTCCCAAGTCTGTTC
*Sox10*	CCAGGTGAAGACAGAGAC	AGACTGAGGGAGGTGTAG
*Sox9*	GCAATACGACTACGCTGAC	ATGTAAGTGAAGGTGGAGTAGA
*Pou3f4*	CTGGAGGAGGCTGATTCAT	GATGGAGGTTCGCTTCTTG
*Pou4f1*	AAACAAATAACCCACACCA AACAG	CTTCCTCAGAGCACCAGTTC
*Ntrk3*	GTGACGAGCGAGGACAATG	GGTAGTAGACAGTGAGAGCAACA
*S100a4*	TGGTCTGGTCTCAACGGTTA	TGGAAGGTGGACACAATTACATC

### Immunofluorescence

Cells were fixed in 4% paraformaldehyde in PBS for 15 min with shaking at room temperature. Inner ear tissues were dissected and fixed in 4% paraformaldehyde in PBS for 2 h with shaking at room temperature, followed by decalcification in 5% EDTA for 4–5 days. Then cells were blocked with 5% heat inactivated horse serum with 0.3% Triton X-100 in PBS for 1 h.

Cells were incubated with primary antibody overnight at 4°C. The primary antibodies used in this study were as follows: anti-Sox2 (goat anti-Sox2;sc-17320, Santa Cruz Biotechnologies; 1:200); anti-Sox10 (rabit anti-Sox10; 69661, Cell Signaling Technology; 1:200); anti-TUJ1 (mouse anti-TUJ1; MMS 435P, Biolegend; 1:2,000); anti-Prox1 (goat anti-Prox1; AF2727, R&D; 1:250); anti-Gata3 (Rabbit anti-Gata3; 5852T, Cell Signaling Technology; 1:1500); anti-Map2 (Mouse anti-Map2; M4403, Sigma-Aldrich; 1:250); anti-Syp (mouse anti-Syp; MA5-14532, Thermofisher; 1:300), and anti-GFP (Rabbit anti-GFP; 31002, Yeasen; 1:400).

Then cells or tissues were incubated with Alexa 488-, Alexa 568-, and/or Alexa 647-labeled secondary antibodies for 1–2 h with shaking at room temperature. Nuclei were visualized with DAPI.

Confocal z-stacks (0.5 μm step size) of cochlear tissues were taken using Leica SP5 microscope equipped with 40× and 63× oil-immersion lens. ImageJ software (version 1.52i, NIH, Bethesda, MD, United States) was used for image processing and three-dimensional reconstruction of z-stacks. All immunofluorescence images shown are representative of at least three individual results. Efficiency of conversion was measured by the number of TUJ1^+^ cells divided by the total number of plated cells from random 6–10 fields. Axon length was measured with NeuronJ, a plugin of ImageJ to facilitate the tracing and quantification of elongated image structures (Ho et al., 2011).

### Molecular Cloning and Lentiviral Infections

cDNAs for Sox2 was cloned into lentiviral constructs of pLKO.1 vector (Addgene 10879). We modified the plasmid to replace the PuroR with Sox2-P2A-EGFP. Lentiviruses were produced by transfection of lentiviral backbones containing the indicated transgenes together with packaging plasmids pSPAX2 (Addgene 12260) and pMD2G (Addgene 12259) into HEK293T cells (ATCC^®^ CRL-3216^TM^). Viruses were concentrated from culture supernatant by ultra-centrifugation (25,000 rpm, 2 h, 4°C). After 24–48 h infection of spheres with 10 μg/ml polybrene in suspension culture, virus-containing medium was replaced with fresh SCM media.

### RNA-seq Analyses

Total RNA was extracted from the tissue using TRIzol^®^ Reagent according the manufacturer’s instructions (TaKara) and genomic DNA was removed using DNase I (TaKara). Then RNA quality was determined by 2100 Bioanalyzer (Agilent) and quantified using the ND-2000 (NanoDrop Technologies). RNA-seq transcriptome library was prepared following TruSeq^TM^ RNA sample preparation Kit from Illumina (San Diego, CA, United States) using 1 μg of total RNA. Libraries were size selected for cDNA target fragments of 200–300 bp on 2% Low Range Ultra Agarose followed by PCR amplified using Phusion DNA polymerase (NEB) for 15 PCR cycles. After quantified by TBS380, paired-end RNA-seq sequencing library was sequenced with the Illumina HiSeq x ten/NovaSeq 6000 sequencer (2 × 150 bp read length). The raw paired end reads were trimmed and quality controlled by SeqPrep and Sickle with default parameters. Then clean reads were separately aligned to reference genome with orientation mode using TopHat (Langmead and Salzberg, 2012) software.

To identify differential expression genes (DEGs) between two different samples, the expression level of each transcript was calculated according to the fragments per kilobase of exon per million mapped reads (FRKM) method. RSEM (Li and Dewey, 2011) was used to quantify gene abundances. R statistical package software EdgeR (Empirical analysis of Digital Gene Expression in R (Robinson et al., 2010) was utilized for differential expression analysis. In addition, functional-enrichment analysis including GO and KEGG were performed to identify which DEGs were significantly enriched in GO terms and metabolic pathways at Bonferroni-corrected *P*-value ≤ 0.05 compared with the whole-transcriptome background.

The transcription data of primary SGN were obtained from previous study (Noda et al., 2018). Raw RNA-seq data have been deposited in the NCBI Gene Expression Omnibus (GEO) under accession number GSE169042.

### Statistical Analysis

Statistical tests were performed using Graphpad Prism 8 (Graphpad Software Inc., La Jolla, CA, United States). Results were reported as mean ± SD. Specific statistical tests used in each experiment were described in figure legends. Results were analyzed using Student’s *t*-test or one-way ANOVA, followed by Bonferroni’s multiple comparisons test.

## Results

### Postnatal Cochlear Spheres Proliferate and Differentiate Into Neuron-Like Cells

**FIGURE 1:**
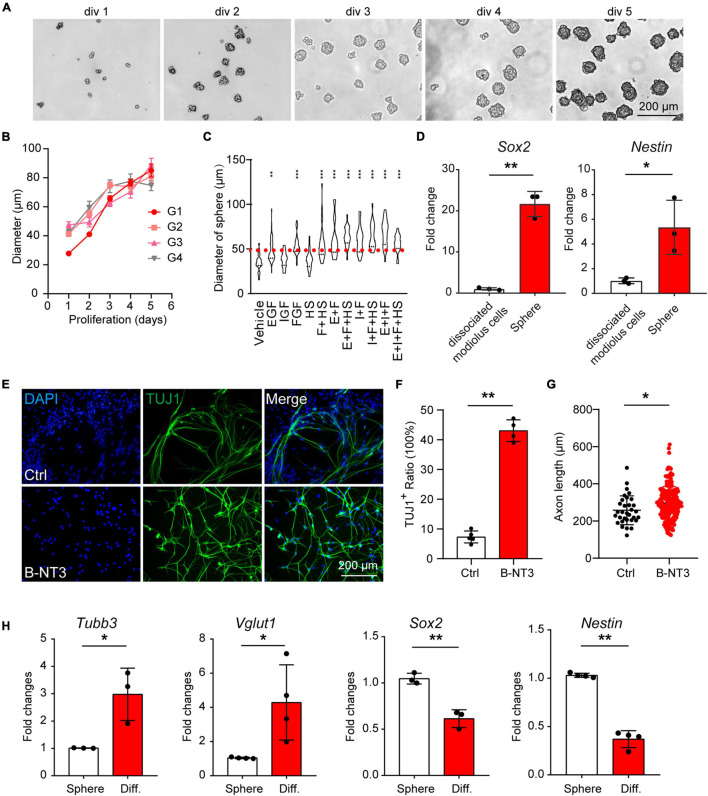
Postnatal cochlear glial cells proliferate as neurospheres and differentiate into neuron-like cells. **(A)** Bright-field images of the proliferating generation 1 (G1) spheres at 1–5 days *in vitro* (div). **(B)** Diameters of G1–G4 spheres during propagation. **(C)** Quantification of sphere diameters at 5 div with supplementations of E, I, F, H. E, EGF; I, IGF; F, FGF, and H, heparan sulfate. *N* = 21–48. *P*-values were calculated against vehicle control. **(D)** mRNA expression of *Sox2* and *Nestin* in cochlear spheres compared to controls (dissociate modiolus cells). *N* = 3, error bars represent mean ± SD. **(E)** Representative TUJ1-positive cells after differentiation at 18 div with BDNF and NT3 (B-NT3). **(F)** The ratio of TUJ1 positive cells. *N* = 5 and 4, error bars represent mean ± SD. **(G)** Axon lengths of the induced neuron-like cells. *N* = 35 and 220, error bars represent mean ± SD. **(H)** mRNA expression of *Tubb3*, *Vglut1*, *Sox2*, and *Nestin* of either spheres or differentiated neuron-like cells (Diff). *N* = 3 or 4, error bars represent mean ± SD. *p < 0.05, **p < 0.01, ***p < 0.001 by one-way ANOVA **(C)** or unpaired student’s *t*-test **(D,F–H)**.

To expand the population of progenitor cells, we dissociated cochlear modiolus from the postnatal 3–4 days (P3–P4) mice and digested into single cells for 3D suspension culture. The cells grew spheres *in vitro* and were able to proliferate for more than five generations (Figures 1A,B). We then optimized the growth media by evaluating the sphere numbers with diameters larger than 50 μm. The result showed that bFGF is the primary factor for spheres growth, consistent with the previous report (Diensthuber et al., 2014a). Heparan sulfate has been reported to promote the binding and activation of FGF (Loo and Salmivirta, 2002). Therefore, we cultured the spheres with serum-free media containing IGF, EGF, FGF, and heparan sulfate (Figure 1C). Real-time quantitative PCR (RT-qPCR) results showed increased expression of neuronal stem cell markers such as *Sox2* and *Nestin* in spheres compared to cochlear modiolus (Figure 1D). These results indicated that postnatal cochlear spheres were able to proliferate and preserve the stemness following passages *in vitro*.

To investigate the neuronal potential of the spheres, we induced the spheres for neuronal differentiation. After induction for 8–9 days, the differentiated cells showed typical bipolar neuronal morphology and neuronal marker TUJ1 expression (Figure 1E, top panels). Brain-derived neurotrophic factor and NT3 are important neurotrophic factors for SGN development and function, and are able to promote neuronal differentiation *in vitro* (Wise et al., 2011; Li et al., 2016; Suzuki et al., 2016; Akil et al., 2019). We found that BDNF and NT3 treatment not only increased the differentiation efficiency (Figures 1E,F) but also promoted neurite extension (Figures 1E,G) during induced differentiation of the spheres. Therefore, BDNF and NT3 were added in the subsequent differentiation experiments. RT-qPCR results also showed that neuronal markers *Tubb3*, *Vglut1* expression were increased, while neuronal stemness markers *Nestin* and *Sox2* were decreased after induced neuronal differentiation (Figure 1H). These results indicate that postnatal cochlear spheres are able to differentiate into neuron-like cells.

### Sox2 Expression Identifies a Subpopulation of Cochlear Glial Cells

Previous studies show the glial cells in association with the SGNs may serve as the source of neuronal progenitors (McLean et al., 2016). Based on the function and localization, cochlear glial cells consist of two major populations including Schwann cells (SCs) and satellite glial cells (SGCs) (Jessen and Mirsky, 2005; Wan and Corfas, 2017). The SCs wrap the axons of SGNs with myelin sheaths and are primarily localized to the osseous spiral lamina (OSL) of the cochlea; while the SGCs are in close association with the SGN cell bodies exclusively localized to the Rosenthal’s canal (RC) of the cochlea (Wan and Corfas, 2017). Despite these differences, the two glial populations share same developmental origin and both serve important roles in survival and function of the SGNs.

**FIGURE 2:**
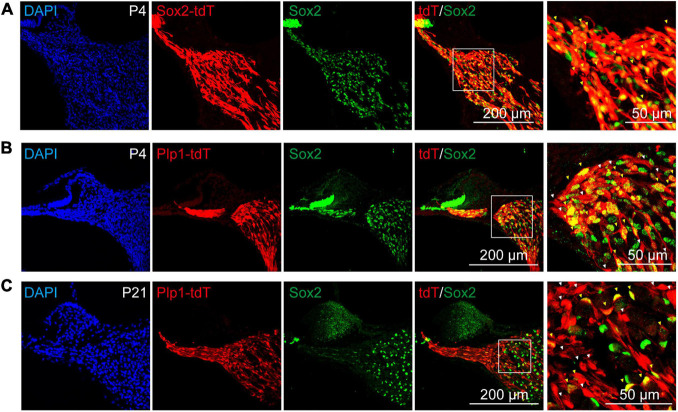
Sox2 labels a subpopulation of cochlear glial cells. **(A)** Immunocytochemical staining of Sox2-tdT cochlea for Sox2, showed Sox2-Cre:tdTomato labeled the Sox2 positive cochlear glial cells in P4 mice. The yellow arrowheads represent Sox2-tdT^+^/Sox2^+^ cells. **(B,C)** Sox2 were expressed in a subpopulation of Plp1-tdT positive cells in both P4 **(B)** and P21 **(C)** cochlea. The white arrowheads represent Plp1-tdT^+^/Sox2^–^ cells, and the yellow arrows represent Plp1-tdT^+^/Sox2^+^ cells. tdTomato expression was induced by tamoxifen from P1 to P3.

It has been reported proteolipid protein 1 (Plp1) was widely expressed in cochlear glial cells, including SCs and SGCs (Jessen and Mirsky, 2005; McLean et al., 2016; Meas et al., 2018b), while Sox2 was expressed in a cochlear glial subpopulation (Zuchero and Barres, 2015; Meas et al., 2018b). To label the cochlear glial cells and the Sox2^+^ subpopulation of glial cells in mice, we crossed the Plp1^CreERT^ and Sox2^CreERT^ with Rosa26-tdTomato line for lineage tracing. After induction with tamoxifen from P1 to P3, majority of the Sox2^+^ cells were labeled with tdTomato (70.6 ± 4.3% of the total Sox2^+^ cells) (Figure 2A). Importantly, Sox2^CreERT^/tdTomato (Sox2-tdT) positive cells all expressed Sox2 in the cochlea, suggesting the inducible Sox2-tdT specifically labels the Sox2^+^ subpopulation of glial cells in mice (Figure 2A).

The results of the immunofluorescence showed that Plp1^CreERT^/tdTomato (Plp1-td) not only labels the Sox2^+^ glial cells, but also Sox2^–^ glial cells at both P4 and P21 (Figures 2B,C), indicating that Sox2 positive cells are a subpopulation of Plp1 positive cochlear glial cells. Thus, Plp1-tdT labels both SCs and SGCs while Sox2-tdT positive glial cells represents a lineage population of cochlear glial cells.

### Cochlear Sox2^+^ Glial Cells Are Primary Progenitors for Neuronal Differentiation

**FIGURE 3:**
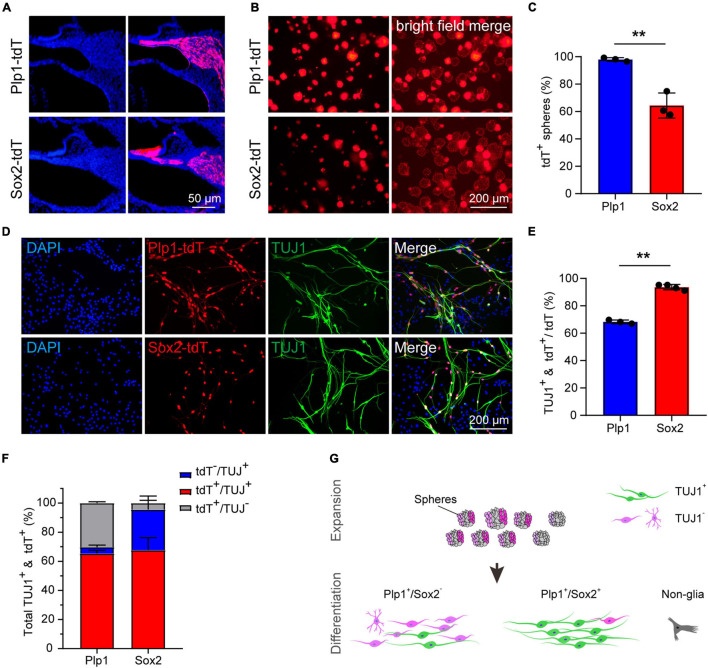
The Sox2^+^ cochlear glial cells were highly efficient in neuronal differentiation. **(A)** Distributions of Sox2-tdT and Plp1-tdT positive cochlear glial cells in P4 cochlea. tdTomato expression was induced by tamoxifen from P1 to P3. **(B,C)** Representative images and quantification of the Sox2-tdT and Plp1-tdT positive spheres at 5 div. *N* = 3, error bars represent mean ± SD. **(D,E)** Representative images and ratios of TUJ1^+^ neurons differentiated from Sox2-tdT and Plp1-tdT positive cells at 8–9 div. *N* = 3 or 4, error bars represent mean ± SD. Data from 3 to 4 independent experiments. **(F)** Percentages of TUJ1^+^ neurons derived from Sox2-tdT or Plp1-tdT positive cells relative to the total amount of TUJ1^+^ plus tdT^+^ cells. **(G)** A model showing Sox2^+^ glial cells exhibit high potential for neuronal differentiation. **p < 0.01 by unpaired student’s *t*-test.

To directly compare the potency of Plp1^+^ and Sox2^+^ glial cells as neuronal progenitors, the lineage-traced cells (Plp1-tdT and Sox2-tdT) were subjected to sphere formation and neuronal differentiation assays (Figure 3A). Although both Plp1-tdT and Sox2-tdT cells were able to form spheres, >95% of the spheres were Plp1-positive while only about 60% of the spheres were Sox2-positive (Figures 3B,C), suggesting that both Plp1^+^/Sox2^+^ and Plp1^+^/Sox2^–^ glial cells were able to proliferate as spheres. Interestingly, Plp1-tdT^+^ or Sox2-tdT^+^ cells were clustered at the periphery of the spheres with the non-glial cells proliferating at the center of spheres (Figure 3B). These non-glial cells were also highly proliferative and Plp1-tdT or Sox2-tdT cells contribute to only about 20 or 12% of the total cells after sphere culture, respectively (Supplementary Figure 1A). To investigate the identity of these non-glial cells, Plp1-tdT^–^ cells were separated from Plp1-tdT^+^ cells by FACS followed by continuous sphere culture (Supplementary Figure 1B). While the non-glial markers failed to express Sox10 or Sox2 as expected, they expressed high levels of Sox9 and Pou3f4 (Supplementary Figure 1C), both of which are enriched in otic mesenchyme cells (Coate et al., 2012; Brooks et al., 2020).

To address if Plp1^+^/Sox2^+^ and Plp1^+^/Sox2^–^ glial cells display different potential for neuronal differentiation, spheres from Plp1-tdT and Sox2-tdT mice were induced to neuronal differentiation for 8–9 days. Firstly, both Plp1-tdT and Sox2-tdT positive cells developed typical bipolar neuronal morphology and expressed neuronal marker TUJ1 (Figure 3D). Intriguingly, while only ∼68% Plp1-tdT cells expressed TUJ1, more than 90% Sox2-tdT cells were co-labeled with TUJ1 (Figure 3E). Further analyses indicated that almost all TUJ1^+^ neurons were derived from Plp1^+^ glial cells but a large proportion of Plp1^+^ cells were unable to differentiate (Figure 3F, left). In contrast, although Sox2^+^ glial cells only contributed to ∼70% of total iNs, almost all Sox2^+^ cells were successfully differentiated (Figure 3F, right). These results indicate that Plp1^+^ cochlear glial cells are the major source of neuronal differentiation *in vitro*, and that Plp1^+^/Sox2^+^ glial subpopulation displays much higher potency/efficiency in neuronal differentiation compared to the Plp1^+^/Sox2^–^ glial cells (Figure 3G).

### P21 Cochlear Sox2^+^ Glial Cells Preserve High Potency of Neuronal Differentiation

**FIGURE 4:**
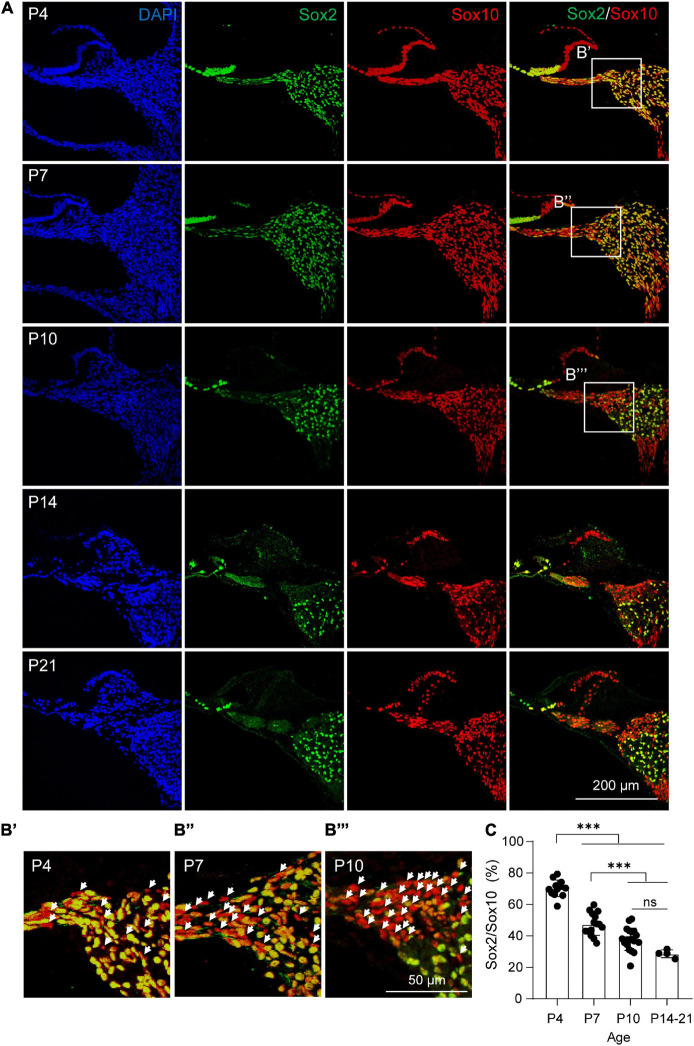
Dynamic changes of Sox2^+^ cochlear glial cells during postnatal development. **(A,B)** Immunocytochemical staining of Sox2 and Sox10 from P4, P7, P10, P14, and P21 cochleae. The white arrow represents the Sox2^–^/Sox10^+^ cell. **(C)** Percentages of Sox2^+^/Sox10^+^ cells from P4, P7, P10, and P14 to P21 cochleae. *N* = 4–13 sections from 3 cochleae, error bars represent mean ± SD. ***p < 0.001 by one-way ANOVA.

As Sox2 expression marks a subpopulation of cochlear glial cells with high neuronal differentiation potency, we next examined the expression of Sox2 at different postnatal ages. The ratio of Sox2^+^ glial cells were calculated based on co-labeling with Sox10, a generic marker of cochlear glial cells similar to Plp1 (Jessen and Mirsky, 2005). We found that the percentage of Sox2^+^ glial cells gradually declined after birth (Figures 4A,B). Specifically, at P4, ∼70% glial cells were Sox2^+^, and the Sox2^+^ ratio decreased to ∼47 and ∼38% at P7 and P10. At hearing onset (P14), the percentage of Sox2^+^ glial cells reduced further to ∼30%, which was maintained at P21 (Figure 4C).

**FIGURE 5:**
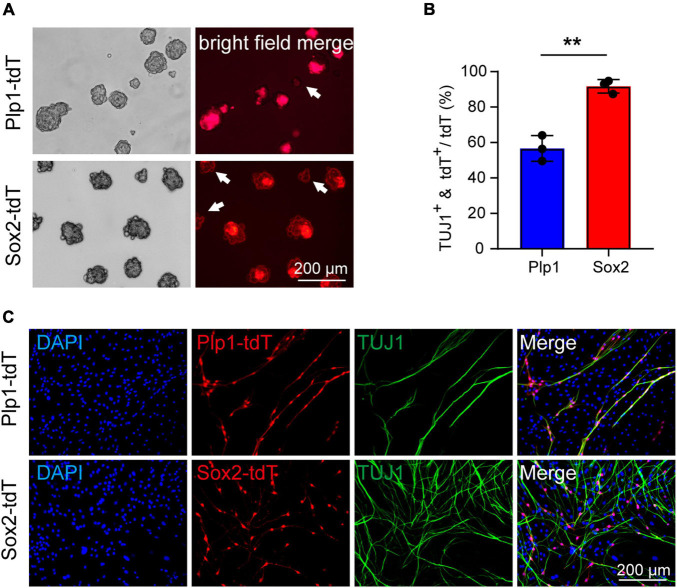
Cochlear Sox2^+^ glial subpopulation preserves high potency of neuronal differentiation in P21 mice. **(A)** Representative images of the Sox2-tdT and Plp1-tdT positive spheres culture 5 div. The white arrow represents the Plp1-tdT or Sox2-tdT negative spheres. **(B,C)** Representative images **(C)** and ratios **(B)** of TUJ1+ neurons differentiated from Sox2-tdT or Plp1-tdT positive cells. *N* = 3, error bars represent mean ± SD. **p < 0.01 by unpaired student’s *t*-test.

We next examined if the Sox2^+^ glial population remains highly efficient in neuronal differentiation at P21. Due to difficulties in obtaining sufficient cochlear glial cells in the calcified modiolus of P21 cochlea, the P21 cochlear glial cells were initially cultured 2D in SCM-FBS medium (Lang et al., 2011) and then transferred to 3D sphere suspension culture. We used the same inducible Plp1^CreERT^ and Sox2^CreERT^ to cross with Rosa26-tdTomato line to specific label the total glial cell population and Sox2^+^ glial cell subpopulation, respectively (Figure 3A). Lineage tracing was induced by tamoxifen injection from P17 to P20, and the cochlear glial cells isolated at P21. Both P21 Plp1-tdT and Sox2-tdT positive cells distributed at the periphery of spheres (Figure 5A), similar to the observation from the P4 spheres (Figure 3B). Followed by induction of neuronal differentiation, ∼55% Plp1^+^ glial cells were TUJ1 positive and ∼90% Sox2^+^ glial cells were TUJ1 positive (Figures 5B,C). In summary, the cochlear Sox2^+^ glial cell subpopulation preserves high potency of neuronal differentiation in both neonatal (P4) and P21 mice.

### Sox2 Downregulation Is Required for Efficient Neuronal Differentiation

**FIGURE 6:**
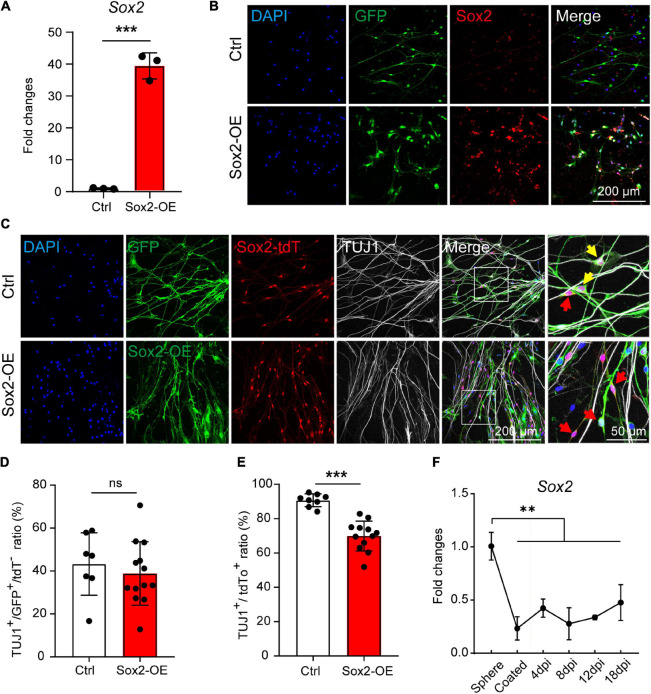
Sox2 overexpression inhibits the neuronal differentiation of Sox2^+^ glial cells. **(A,B)** mRNA expression **(A)** and immunocytochemical staining **(B)** of Sox2 overexpressed in cochlear glial cells. *N* = 3, error bars represent mean ± SD. **(C)** Immunocytochemical staining of TUJ1^+^ neurons differentiated from cochlear glial cells of Sox2-tdT mice. The yellow arrows represent TUJ1^+^/GFP^+^/tdT^+^ cells, and the red arrows represent TUJ1^–^/GFP^+^/tdT^+^ glial cells. **(D)** Quantification of TUJ1^+^/GFP^+^ cells from Sox2-tdT negative glial cells. *N* = 8–13 images from 3 coverslips, error bars represent mean ± SD. **(E)** Quantification of TUJ1^+^/Sox2-tdT^+^ cells from glial cells overexpressing either GFP or Sox2. *N* = 7–11 images from 3 coverslips, error bars represent mean ± SD. **(F)** mRNA expression of *Sox2* during induced neuronal differentiation of the cochlear glial cells at different conditions and time points. *N* = 3, error bars represent mean ± SD. **p < 0.01, ***p < 0.001 by one-way ANOVA **(F)** or unpaired student’s *t*-test **(A,E)**.

To further investigate if the Sox2 expression contributes to the high neuronal differentiation potential of the Sox2^+^ glial cells, we overexpressed Sox2 in both Sox2^+^ and Sox2^–^ glial cells during 3D suspension culture of the spheres. Spheres were infected with lentivirus encoding Sox2-GFP or GFP control and the Sox2 overexpression was validated by both RT-qPCR and immunofluorescence (Figures 6A,B).

Next, the spheres were induced to neuronal differentiation for 8–9 days and were immuno-stained with TUJ1 antibody. We first evaluated the effect of Sox2 overexpression on neuronal differentiation of Sox2^–^ glial cells. Neuronal differentiation of Sox2^–^ glial cells, as marked by TUJ1^+^/Sox2-tdT^–^ cells, was not affect by Sox2 overexpression compared to GFP controls (Figures 6C,D). This result suggest that the incompetence of Sox2^–^ glial cells in neuronal differentiation is not due to lack of Sox2 expression. To our surprise, neuronal differentiation of the Sox2-tdT (Sox2^+^) glial cells was significantly reduced after Sox2 overexpression (Figures 6C,E). Consistently, *Sox2* expression was decreased dramatically from the proliferative stage in 3D culture to the differentiation stage in 2D culture, and then maintained at a stably low level during the entire process of neuronal differentiation (Figure 6F). These results suggest that Sox2^+^ glial cells exhibit high potential for neuronal differentiation independent of Sox2 expression, and that downregulation of Sox2 is required for efficient neuronal differentiation.

### Small Molecules Promote Neuronal Differentiation and Maturation Toward Spiral Ganglion Neuron Fate

Although cochlear glial cells can be differentiated into iNs, the specific markers for SGNs were rarely detected, indicative a major huddle in SGN fate conversion and maturation. Small molecules have been reported to promote neuronal reprogramming from somatic and glial cells (Hu et al., 2015; He et al., 2017; Belin-Rauscent et al., 2018). To promote the neuronal differentiation and maturation to SGNs from cochlear glial cells, we next screened 10 neurogenic small molecules that were shown to activate neuronal signaling pathways, inhibit glial signaling pathways, or modulate epigenetics to promote neuronal reprogramming. Small molecules selected for our initial screening were as follows: SB431542, CHIR99021, Y27632, retinoic acid (RA), valproic acid (VPA), Forskolin, ISX9, I-BET151, Vitamin C (Vc), and LIF.

It has been reported that Forskolin, ISX9, I-BET151, and CHIR99021 (FIBC) combination induced neuronal differentiation from mouse embryonic fibroblasts (MEFs) and astrocytes efficiently (Hu et al., 2015; He et al., 2017). Firstly, we used the FIBC combination to test the concentration for neuronal differentiation from cochlear glial cells (Supplementary Figure 2A). Based on the bipolar neuronal morphology of TUJ1^+^ cells and minimal cytotoxicity, we identified the optimal concentrations of Forskolin (20 μM), ISX9 (20 μM), I-BET151 (2 μM), and CHIR99021 (10 μM). Next, we induced the neuronal differentiation with FIBC in the presence of SB431542, Y27632 (Y), RA, VPA, Vc, or LIF (L). The results showed FIBC and Y27632 (FIBCY) and FIBC and LIF (FIBCL) increased the expression of Prox1 (Supplementary Figure 3E), an SGN marker, compared with other small molecules (Supplementary Figure 2B). Thus, we used FIBCY and FIBCL as the small molecules cocktail for neuronal induction and maturation.

**FIGURE 7:**
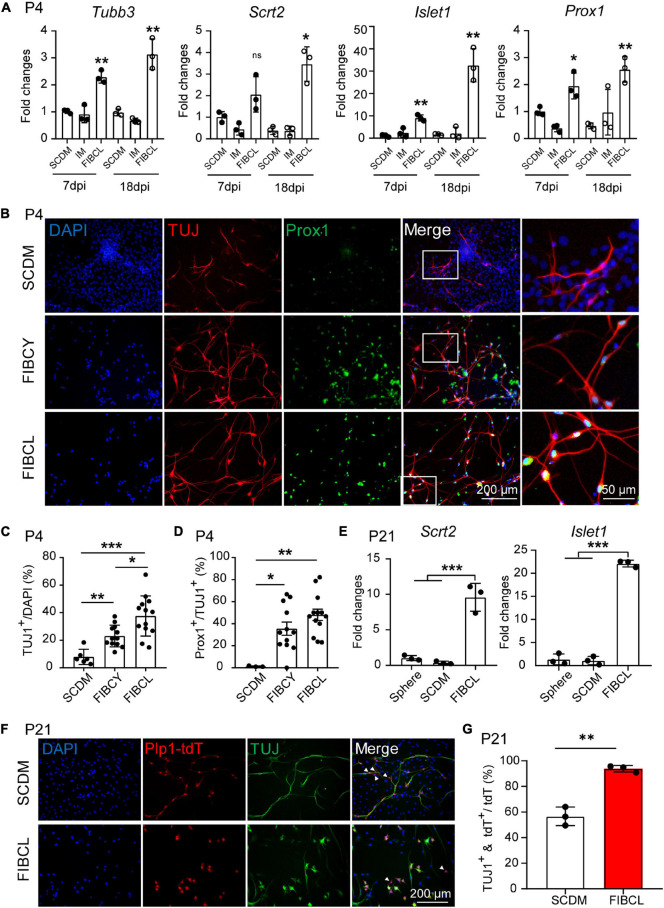
Small molecules promote neuronal differentiation and maturation of cochlear glial cells. **(A)** RT-qPCR gene expression analyses of P4 cochlear glial cells differentiated in SCDM, IM or FIBCL at 7 and 18 div. FIBCL, Forskolin, ISX9, I-BET151, CHIR99021, and LIF. *N* = 3, error bars represent mean ± SD. **(B)** Immunocytochemical stainings of TUJ1 and Prox1 in neurons induced from P4 cochlear glial cells at different conditions. **(C,D)** Percentages of TUJ1 positive cells **(C)** and TUJ1/Prox1 double positive cells **(D)** differentiated in SCDM, FIBCY, or FIBCL. *N* = 3–13 images from 2 wells of each condition, error bars represent mean ± SD. **(E)** mRNA expression of SGN markers *Scrt2* and *Islet1*. *N* = 3, error bars represent mean ± SD. **(F)** Immunocytochemical staining of TUJ1 of induced neurons from P21 Plp1-tdT cochlear glial cells. The white arrowheads represent the Plp1-tdT^+^/TUJ1^–^ cells. **(G)** Percentages of TUJ1 positive cells differentiated from P21 Plp1-tdT^+^ cochlear glial cells. *N* = 3, error bars represent mean ± SD. *p < 0.05, **p < 0.01, ***p < 0.001 by one-way ANOVA **(A,C–E)** or unpaired student’s *t*-test **(G)**.

RT-qPCR results also showed increased expression of generic neuronal markers such as *Tubb3* (Supplementary Figures 3A,B), and SGN-specific markers including *Prox1*, *Islet1*, and *Scrt2* (Supplementary Figure 3G) of the iNs by small molecules FIBCL compared with SCDM or IM control medium (Figure 7A). Furthermore, the expression of *Tubb3*, *Scrt2*, and *Islet1* were further increased in a temporal manner during neuronal differentiation (Figure 7A, 7 dpi vs. 18 dpi). FIBCY and FIBCL increased the number of TUJ1^+^ cells and induced both co-labeling of TUJ1 and Prox1 (Figure 7B). Quantitative analyses indicate that FIBCY and FIBCL significantly increased the percentage of overall TUJ1^+^ cells (Figure 7C) and percentage of TUJ1^+^ cells co-expressing Prox1 (Figure 7D). FIBCL appeared to perform better in inducing TUJ1^+^ iNs than FIBCY (Figure 7C). These results suggested that FIBCY/FIBCL promoted neuronal differentiation and maturation toward SGNs from neonatal cochlear glial cells.

We then induced neuronal differentiation of P21 Plp1-tdT glial cells in the presence or absence of FIBCL. For P21 glial cells, FIBCL also promoted the expression of SGN markers *Scrt2*, *Islet1* (Figure 7E). Remarkably, FIBCL treatment resulted in a significant increase in the percentage of TUJ1^+^/Plp1-tdT^+^ cells (∼90%) compared to the control group (∼55%) (Figures 7F,G). As Plp1^+^/Sox2^+^ but not Plp1^+^/Sox2^–^ cells showed potent neuronal differentiation in control condition, FIBCL treatment may have also promoted neuronal differentiation of Plp1^+^/Sox2^–^ glial cells. Together, these results highlight the effectiveness of small molecule combinations FIBCY and FIBCL in promoting neuronal differentiation and SGN maturation of neonatal and P21 cochlear glial cells.

### FIBCL-Induced Neurons Display Similar Transcriptomic Profile as the Primary Spiral Ganglion Neurons

**FIGURE 8:**
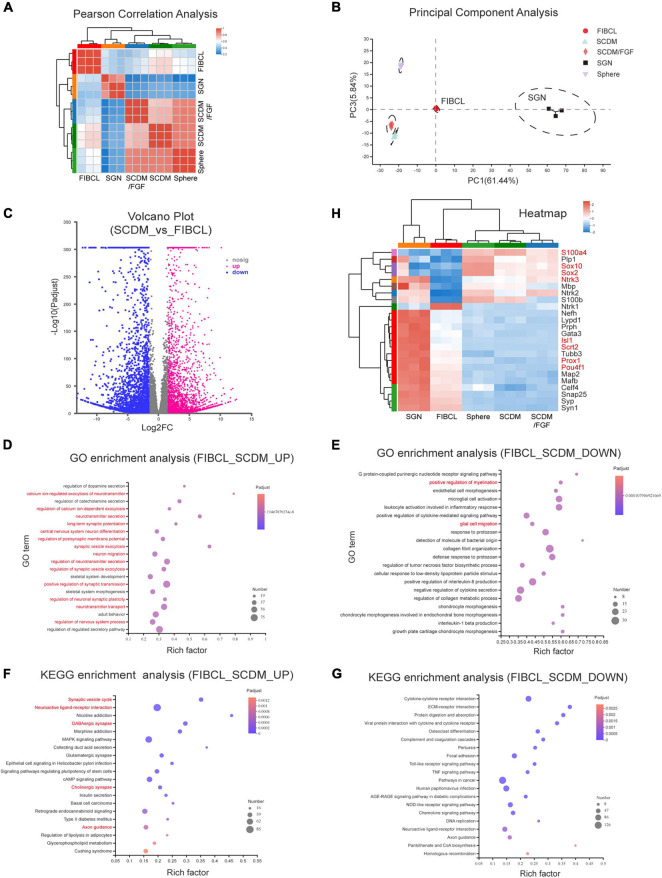
Transcriptomic analyses of the induced neurons. **(A,B)** Pearson correlation analysis **(A)** and principal component analysis **(B)** of neurospheres, differentiated culture in SCDM, SCDM/FGF, FIBCL media, and primary SGNs. **(C)** Volcano graph of the genes upregulated and downregulated in the FIBCL-induced neurons compared to the SCDM media. **(D,E)** GO enrichment analysis of upregulated **(D)** and downregulated **(E)** functional pathways in the FIBCL-induced neurons compared to the SCDM media. **(F,G)** KEGG enrichment analysis of upregulated **(D)** and downregulated **(E)** cellular processes in the FIBCL-induced neurons compared to the SCDM media. **(H)** Heatmap graph of specific glial and neuronal genes expressed in spheres, differentiated culture in SCDM, SCDM/FGF, FIBCL media, and primary SGNs. Changes in expression of selected genes (gene symbol highlighted in red) were validated by RT-qPCR in Supplementary Figure 4.

To evaluate the maturity under different induction conditions of iNs, we performed transcriptome sequencing analyses. Pearson correlation analyses showed that the expression profiles of neurons induced by SCDM and SCDM/FGF were relatively closer to that of the spheres (Figure 8A). However, the overall expression profile of FIBCL-iNs was distinct from those of the spheres, SCDM or SCDM/FGF, and was more similar to that of the primary SGNs (Figure 8A). In addition, the results of principal component analyses (PCA) showed that FIBCL-iNs were similar to the SGNs (Figure 8B). Comparing the differentially expressed genes between SCDM and FIBCL-iNs, we found about 3200 genes in the FIBCL group were significantly upregulated and 3000 genes were significantly downregulated (Figure 8C). The GO and KEGG enrichment analyses of these genes, respectively showed that the upregulated genes were mainly enriched in neuron development, neurotransmitter, synapse and calcium ion channels; while the downregulated genes contained glial cell migration and myelination, as well as GPCR and cytokine signaling pathways (Figures 8D–G).

Analyses of specific gene expression revealed that glial cell-specific genes such as *Plp1*, *Sox10*, *S100*β, and *MBP* still maintained high expression in sphere, SCDM and SCDM/FGF groups, but the expression levels of these genes were significantly downregulated in FIBCL-iNs group (Figure 8H). Interestingly, Sox2 expression was also further reduced after FIBCL treatment (Figure 8H), consistent with an inhibitory role of Sox2 in neuronal differentiation (Figure 6). In addition, expression levels of neuron-specific genes such as *Tubb3*, *Nefh*, *Snap25*, *Syp* (Supplementary Figure 3D), and *Map2* (Supplementary Figure 3F) were induced in SCDM and SCDM/FGF groups compared to the spheres, which were further upregulated in the FIBCL group (Figure 8H). Finally, the expression levels of SGN-specific genes such as *Isl1*, *Pou4f1*, *Prox1*, *Gata3* (Supplementary Figure 3C), and *Mafb* were also significantly upregulated, while cochlear glial genes such as *Sox10*, *Sox2*, and *S100a4* were downregulated in the FIBCL-iNs (Figure 8H and Supplementary Figure 4). Overall, the RNA-seq results indicate that iNs in control medium were at the immature state, retaining some of the glial cell characteristics; while small molecules FIBCL removed glial cell barriers and further promoted neuronal maturation.

## Discussion

**FIGURE 9:**
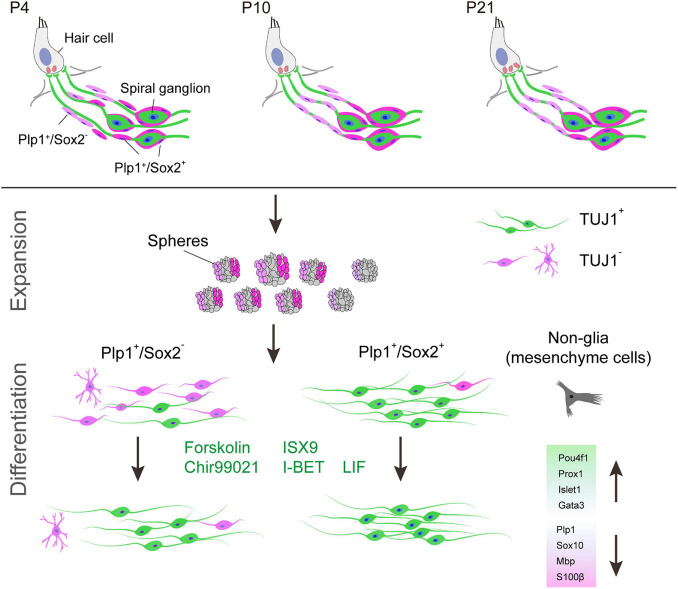
Graphic summary of cochlear Sox2^+^ glial cells as potent progenitors for spiral ganglion neuron reprogramming induced by small molecules. Sox2^+^ cells are a subpopulation of Plp1^+^ cochlear glial cells. Although both Sox2^+^ and Sox2^–^ glial cells can form neurospheres, Plp1^+^/Sox2^+^ spheres are more neurogenic compared to Plp1^+^/Sox2^–^ spheres. Efficiency and maturity of the induced neurons can be further improved by a small molecule cocktail.

Spiral ganglion neurons lack the ability to regenerate after damage in the mammalian cochlea, which is a major cause of auditory neuropathy and may compromise the therapeutic effects of cochlear implants (Guo et al., 2019). Extensive studies have been performed using neural stem cells for regeneration of neurons and the SGNs (Fang et al., 2019; Li et al., 2019; Tang et al., 2019; Han et al., 2020; Xia et al., 2020; Yang et al., 2020, 2021; Yuan et al., 2021). Yet, functional regeneration of SGNs from the resident glial cells may represent a novel strategy for hearing restoration caused by SGN damages. In this study, we found that the cochlear Sox2^+^ glial cell subpopulation exhibits high potency of neuronal differentiation, and the efficiency of neuronal differentiation requires Sox2 downregulation. Furthermore, we identified a small molecule combination that promotes neuronal differentiation and maturation toward SGN fate (Figure 9).

### Cochlear Sox2^+^ Glial Cell Subpopulation as Potent Neuronal Progenitors

The starting progenitor population is one of the key considerations for successful cell fate reprogramming (Gascon et al., 2017). In the brain and retina, astrocytes (Guo et al., 2014; Qian et al., 2020) and Müller glial cells (Qian et al., 2020) have been shown to convert to new neurons and retinal ganglion cells under specific conditions. Cochlear glial cells were regarded as specialized cells and exhibited characteristics as well as corresponding functions of glial cells. Similar to the central glial cells, cochlear glial cells can also proliferate and regenerate themselves upon injury *in vivo* (Lang et al., 2015; Wan and Corfas, 2017), suggesting that these glial cells may also function as progenitors for SGNs.

Cochlear glial cells are heterogeneous and include SCs and Satellite cells (Wan and Corfas, 2017). While Plp1 and Sox10 are generic markers for cochlear glial cells, Sox2 is only expressed in a subset of glial cells. Sox2^+^ glial cells are mainly located around the SGN cell body, which overlapped with SGCs. A few Sox2^+^ glial cells are also located along SGN axons at OSL after P14 in mice. In this study, we identified Sox2^+^ glial cells sub-population as potent neuronal progenitors compared to Sox2^–^ glial cells, suggesting that Sox2^+^ glial cells may be the target progenitor population for SGN regeneration in future studies.

In recent years, mechanisms of proliferation and neuronal differentiation of the cochlear glial progenitor has been under close investigation. The Plp1^+^ cochlear glial cells serve as potent progenitors for neurons, astrocytes and oligodendrocytes, but not hair cells *in vitro* (McLean et al., 2016). Cochlear glial cells isolated from Sox2-eGFP reporter mice also displayed potent neurogenic potential *in vitro* (Lang et al., 2015; Meas et al., 2018a). However, a direct comparison of glial subpopulations is lacking and the iNs did not appear to mature as SGNs. Importantly, two groups recently reported that iNs can be generated from Plp1^+^ glial cell by Ng1/Nd1 or Lin28 overexpression *in vivo* post-SGN injury, and that iNs expressed both pan-neuronal and SGN markers (Kempfle et al., 2020; Li X. et al., 2020). However, the efficiency of neuronal conversion and neuronal maturity is still limited. Based on our findings, we believe that direct reprogramming of the Sox2^+^ glial cells may be key to enhance functional glia-to-neuron conversion *in vivo*.

### Role of Sox2 Expression in Neuronal Differentiation From Cochlear Glial Cells

Developmentally, Sox2 is generally expressed in a variety of cells types including neuronal stem cells (Graham et al., 2003; Zhang and Cui, 2014; Cui et al., 2018) and tissue specific cells, such as DRG satellite cells and cochlear supporting cells (Kiernan et al., 2005; Koike et al., 2015). Our finding on higher potential of Sox2^+^ glial cells poses a question on whether Sox2 expression is required for efficient neuronal induction or merely serves as a marker for progenitors with high neurogenic potential. Our results point to the later scenario and may also suggest that Sox2 serves as an inhibitory signal for efficient neuronal induction. This notion is consistent with the observations in CNS, whereby Sox2 was highly expressed in neuronal progenitor cells (NPCs) and inhibited the neuronal differentiation of these progenitor cells (Graham et al., 2003; Cavallaro et al., 2008). Sox2 expression was gradually downregulated during the process of neuronal differentiation (Cavallaro et al., 2008; Cui et al., 2018; Mercurio et al., 2019). Knockdown of Sox2 partially rescued the impairment of neuronal differentiation induced by miR-145 downregulation (Morgado et al., 2016).

Intriguingly, although Sox2 was downregulated during neuronal differentiation of the cochlear glial cells, we observed that the iNs still maintained the expression of Sox2 at a specific level. Sox2 expression of iNs was higher than that of SGNs, which may present a barrier for further neuronal maturation. This is in congruence with our finding that the small molecule cocktail promoted further neuronal maturation while also significantly reduced the expression of Sox2.

### Small Molecules Promote Efficiency and Maturity of Induced Neurons

Neuronal reprogramming may not be initiated due to the stable barriers of glial cells identity and lack of neuronal factors to regulate specific transcriptional program (Mertens et al., 2016; Black and Gersbach, 2018; Li et al., 2018). Small molecules that modulate specific signaling pathways have been shown to promote neuronal reprogramming. For example, small molecules CHIR99021, Forskolin, and ISX9 were shown to improve the neuronal conversion and differentiation (Schneider et al., 2008; Dworkin and Mantamadiotis, 2010; Liu et al., 2013; Li et al., 2015; Gao et al., 2017; Gascon et al., 2017; Yang et al., 2019). IBET151 serves to erase the initial cell-fate specific gene expression pattern (Di Micco et al., 2014; Li et al., 2015; Wu et al., 2015; Marazzi et al., 2018). VPA has been reported to enhanced neuronal induction of cochlear glial cells (Moon et al., 2018). However, more small molecules were hardly studied in cochlear glia-to-neuron induction.

In this study, the small molecule cocktail we identified can promote neuronal maturation and cell fate conversion toward SGNs. The results showed upregulation of generic neuronal genes and SGN specific genes such as *Prox1*, *Islet1*, *Pou4f1*, and *Scrt2*. Consistent with previous reports that Ng1/Nd1 or Lin28 induced glial cells-to-auditory neuron conversion in the cochlea (Kempfle et al., 2020; Li X. et al., 2020), the iNs also exhibited increased expression of both *Nd1* and *Lin28*. Furthermore, we observed the downregulation of glial cell markers such as Plp1, Sox10, S100β, and MBP. Thus, the small molecule cocktail induces neuron differentiation and maturation by removal of glial identity and establishment of neuronal identity.

### Outlook Into Functional Maturation of the Induced Neurons

Although the small molecule cocktail greatly promoted neuronal induction toward SGN, expression levels of SGN-specific genes were still lower than those in primary SGNs and some key SGN markers, such as *Ntrk3*, were not upregulated. It is possible that means to upregulate these developmental and functional relevant genes may further promote the maturation of the iNs to functional SGNs *in vivo*.

Other challenges in functional maturation of the iNs remain to be addressed. For example, synaptic connections between the iNs and hair cells or cochlear nucleus are the fundamental elements of the cochlear neuronal circuitry *in vivo*. Previous studies showed synapse formation between ESC-derived iNs and sensory epithelium or cochlear nucleus *in vitro* with limited efficiency (Liu et al., 2018; Meas et al., 2018b) and the *de novo* synapses were not observed *in vivo* (Kempfle et al., 2020; Li X. et al., 2020). Secondly, SGNs are heterogenous in nature and display distinct molecular signature and spontaneous firing rates (Petitpre et al., 2018). The determination of SGN identity is induced by interaction of different spontaneous activity from hair cells (Petitpre et al., 2018; Shrestha et al., 2018; Sun et al., 2018). How the iNs may adopt specific SGN subtype and integrate into the cochlear neuronal circuitry needs to be explored in future studies.

## Data Availability Statement

The datasets presented in this study can be found in online repositories. The names of the repository/repositories and accession number(s) can be found in the article/Supplementary Material.

## Ethics Statement

The animal study was reviewed and approved by Institutional Animal Care and Use Committee of Model Animal Research Center of Nanjing University.

## Author Contributions

GW conceived the study. ZC and GW designed the experiments, analyzed the results, and wrote the manuscript. ZC, YH, CY, QL, and CQ performed the experiments. All authors have read and agreed to the published version of the manuscript.

## Conflict of Interest

The authors declare that the research was conducted in the absence of any commercial or financial relationships that could be construed as a potential conflict of interest.

## Publisher’s Note

All claims expressed in this article are solely those of the authors and do not necessarily represent those of their affiliated organizations, or those of the publisher, the editors and the reviewers. Any product that may be evaluated in this article, or claim that may be made by its manufacturer, is not guaranteed or endorsed by the publisher.

## Supplementary Material

Supplementary Figure 1 The non-glial cells in the spheres are likely derived from cochlear mesenchyme cells. **(A)** Percentage of Plp1-tdT^+^ or Sox2-tdT^+^ cells to the total number of cells after sphere culture. *N* = 8–10, error bars represent mean ± SD. **(B)** Representative images of Plp1-tdT^+^ glial and Plp1-tdT^–^ non-glial cells after FACS followed by sphere culture. **(C)** RT-qPCR analyses of the glial and non-glial spheres with glial markers (Sox10, Sox2) and mesenchymal markers (Sox9, Pou3f4). *N* = 3, error bars represent mean ± SD. ****p* < 0.001 by unpaired student’s *t*-test.

Supplementary Figure 2 Small molecules screening and optimization. **(A)** Representative images of TUJ1 immunostaining of iNs treated with various concentrations of small molecules, I-BET, Forskolin, ISX9, Chir99021 at 15 div. **(B)** Representative images of Prox1 immunostaining of iNs treated with small molecules Y27632, SB431542, RA, LIF, Vitamin C, and VPA at 15 div. The white arrowheads represent Prox1^+^ cells.

Supplementary Figure 3 Specific expression of SGN markers. **(A)** Representative cartoon showing cochlear localization of SGNs and surrounding glial cells. **(B–G)** Specific expressions of TUJ1 **(B)**, Gata3 **(C)**, Syp **(D)**, Prox1 **(E)**, Map2 **(F)**, and Scrt2 **(G)** in P3–P42 cochlear SGNs.

Supplementary Figure 4 RT-qPCR validations of glial and neuronal genes regulated during FIBCL-induced differentiation. mRNA expressions of *Islet1*, *Scrt2*, *Pou4f1*, *Prox1*, *Sox10*, *Sox2*, *Ntrk3*, and *S100a4* (as highlighted in Figure 8H) in non-differentiated spheres or differentiated cultures treated with SCDM, SCDM/FGF, or FIBCL were analyzed by RT-qPCR. *N* = 3, error bars represent mean ± SD. **p* < 0.05, ***p* < 0.01, ****p* < 0.001 by one-way ANOVA.
