# Supplementary figures and images for: Cochlear Sox2+ Glial Cells Are Potent Progenitors for Spiral Ganglion Neuron Reprogramming Induced by Small Molecules
Source: Front Cell Dev Biol. 2021 Sep 21;9:728352. doi: 10.3389/fcell.2021.728352 (PMC8490772; doi:10.3389/fcell.2021.728352)

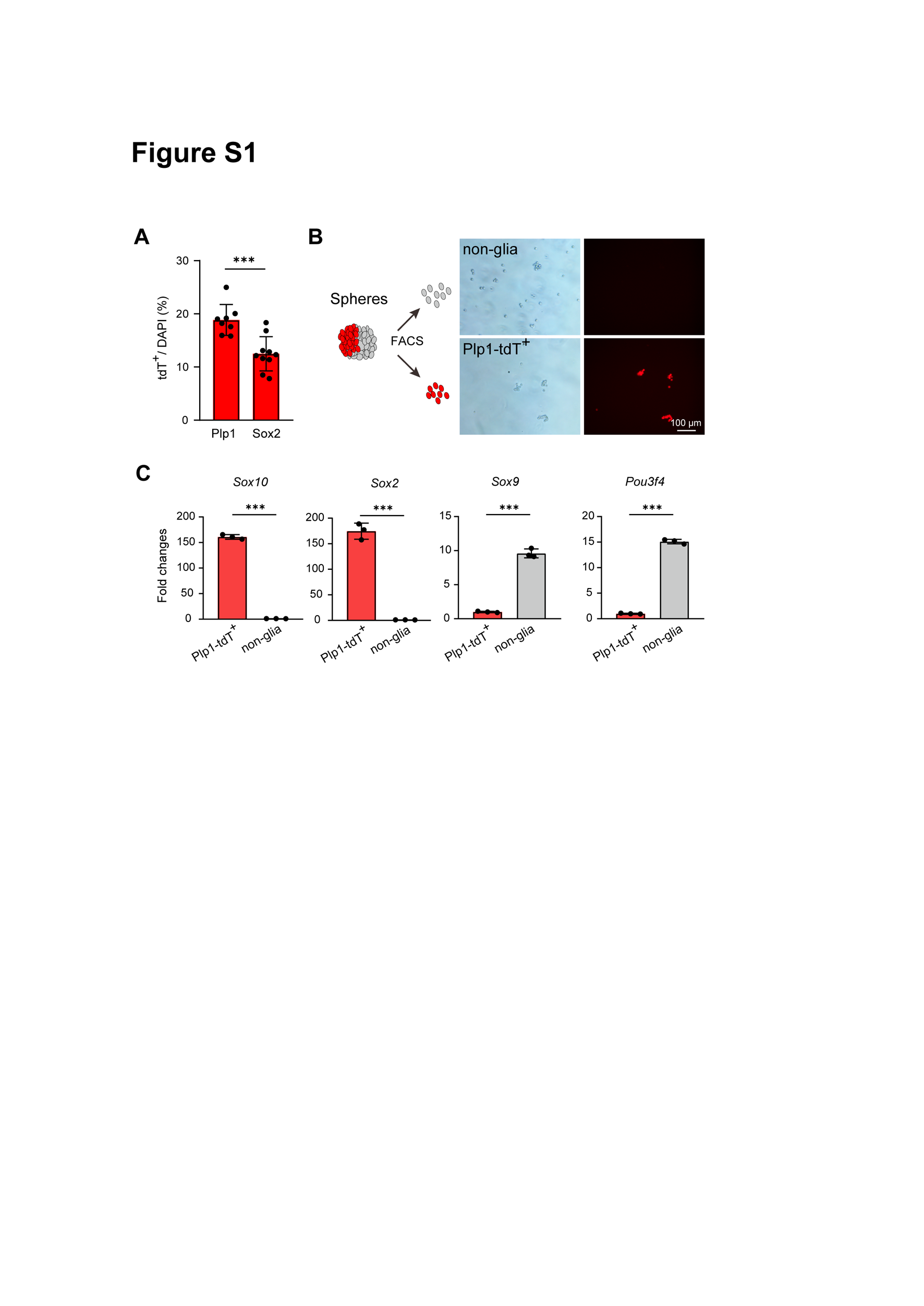

Supplement: Supplementary Figure 1 — The non-glial cells in the spheres are likely derived from cochlear mesenchyme cells. (A) Percentage of Plp1-tdT+ or Sox2-tdT+ cells to the total number of cells after sphere culture. N = 8–10, error bars represent mean ± SD. (B) Representative images of Plp1-tdT+ glial and Plp1-tdT– non-glial cells after FACS followed by sphere culture. (C) RT-qPCR analyses of the glial and non-glial spheres with glial markers (Sox10, Sox2) and mesenchymal markers (Sox9, Pou3f4). N = 3, error bars represent mean ± SD. ***p < 0.001 by unpaired student’s t-test. [file Image_1.TIF]

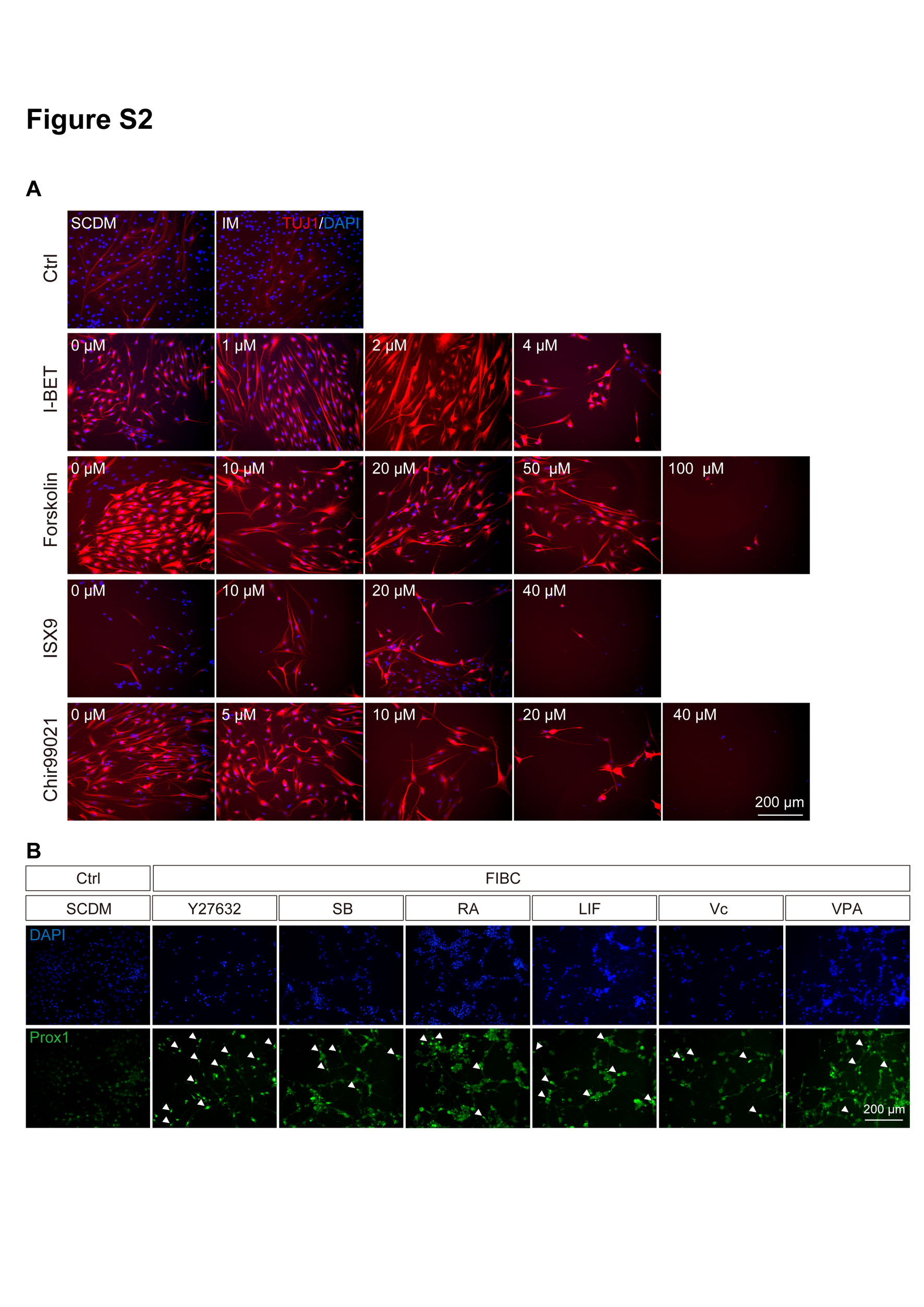

Supplement: Supplementary Figure 2 — Small molecules screening and optimization. (A) Representative images of TUJ1 immunostaining of iNs treated with various concentrations of small molecules, I-BET, Forskolin, ISX9, Chir99021 at 15 div. (B) Representative images of Prox1 immunostaining of iNs treated with small molecules Y27632, SB431542, RA, LIF, Vitamin C, and VPA at 15 div. The white arrowheads represent Prox1+ cells. [file Image_2.TIF]

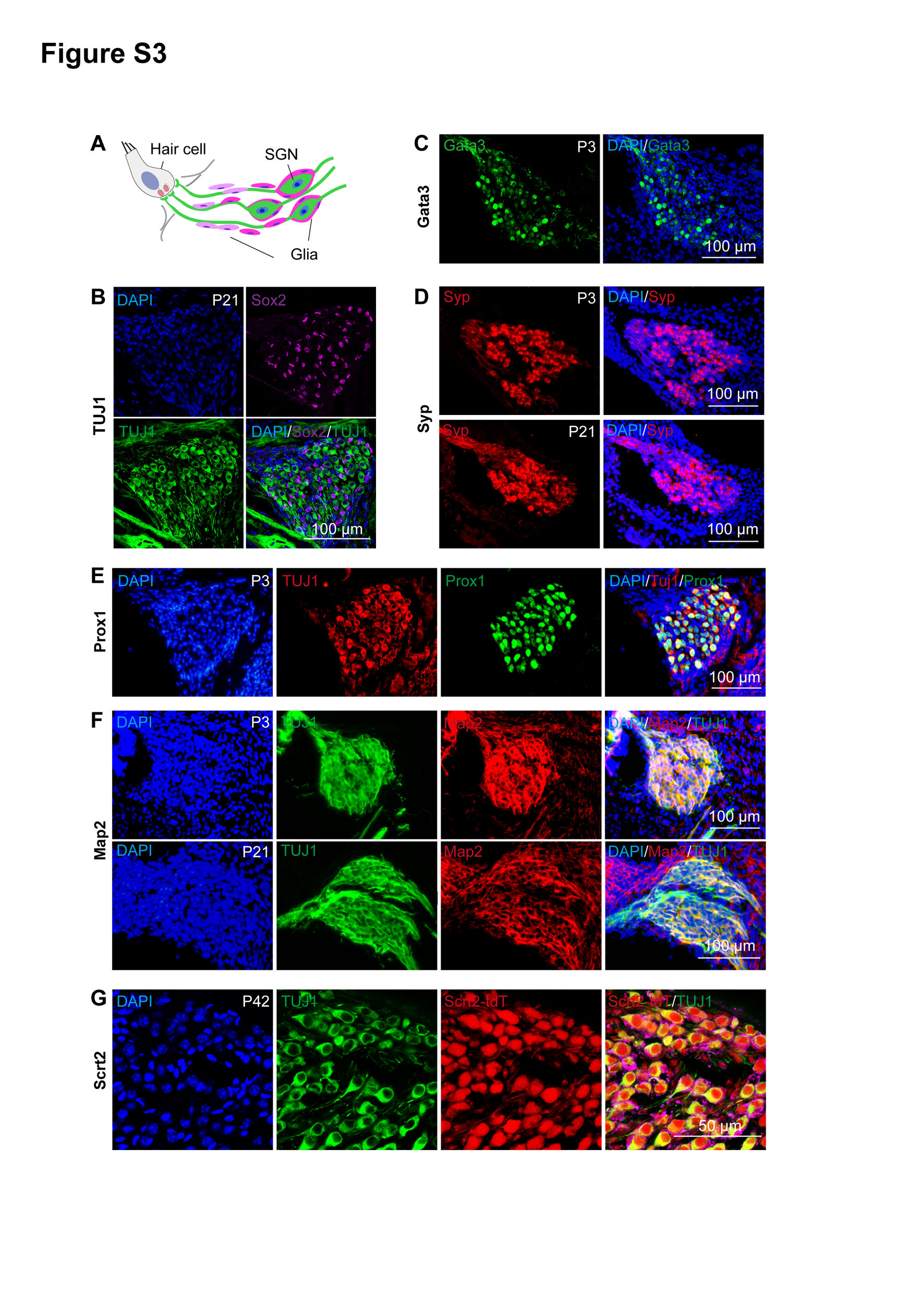

Supplement: Supplementary Figure 3 — Specific expression of SGN markers. (A) Representative cartoon showing cochlear localization of SGNs and surrounding glial cells. (B–G) Specific expressions of TUJ1 (B), Gata3 (C), Syp (D), Prox1 (E), Map2 (F), and Scrt2 (G) in P3–P42 cochlear SGNs. [file Image_3.TIF]

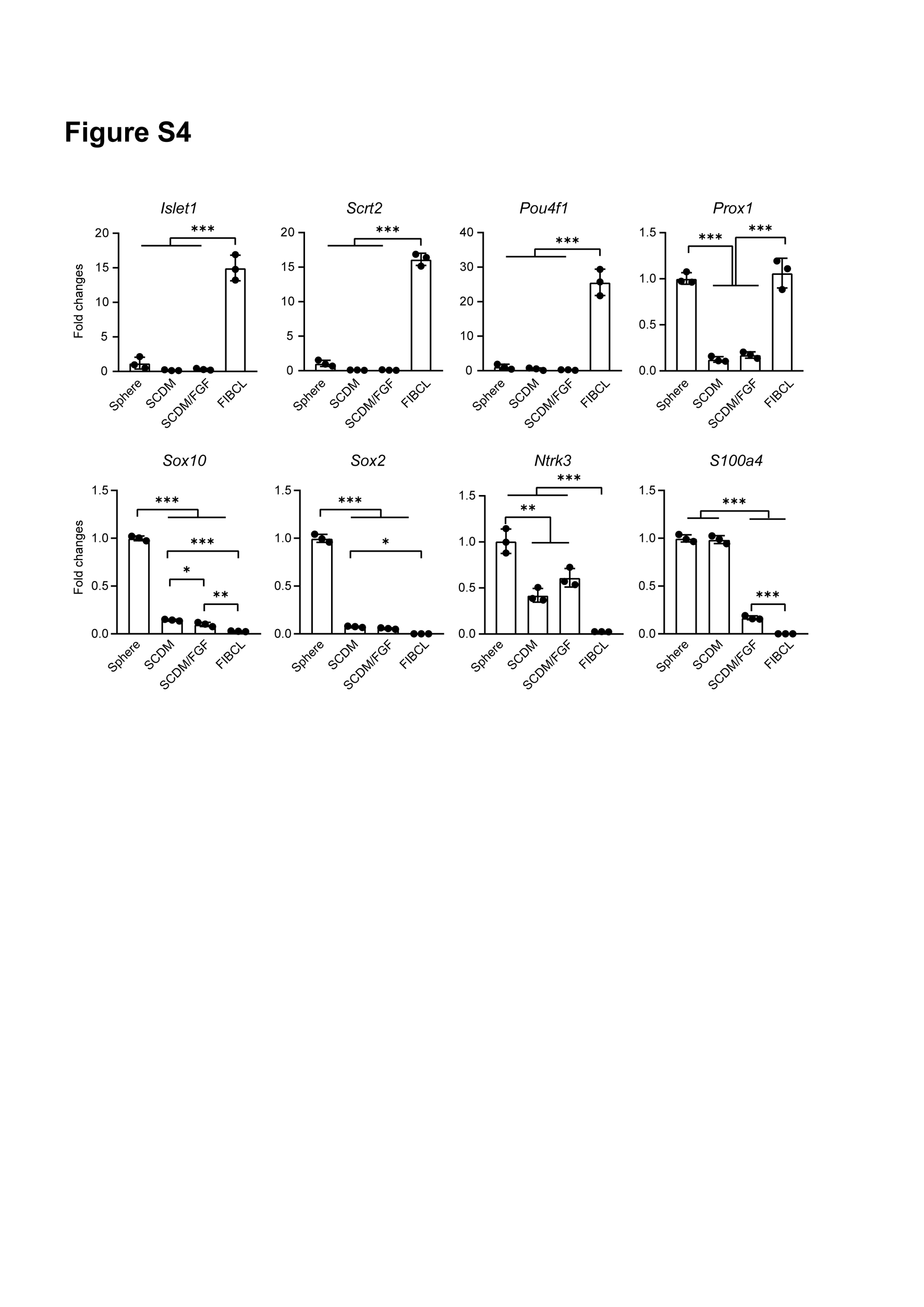

Supplement: Supplementary Figure 4 — RT-qPCR validations of glial and neuronal genes regulated during FIBCL-induced differentiation. mRNA expressions of Islet1, Scrt2, Pou4f1, Prox1, Sox10, Sox2, Ntrk3, and S100a4 (as highlighted in Figure 8H) in non-differentiated spheres or differentiated cultures treated with SCDM, SCDM/FGF, or FIBCL were analyzed by RT-qPCR. N = 3, error bars represent mean ± SD. *p < 0.05, **p < 0.01, ***p < 0.001 by one-way ANOVA. [file Image_4.TIF]
